# Comparative Analysis of Meat Quality and Flavor-Forming Volatile Compounds in Longissimus Dorsi from Different Beef Breeds

**DOI:** 10.3390/vetsci13050454

**Published:** 2026-05-06

**Authors:** Juan Xu, Qian Li, Huibin Zhang, Shuanping Zhao, Hai Jin, Qinggang Li, Xinyi Du, Sihua Jin, Lei Xu

**Affiliations:** 1 Anhui Province Key Laboratory of Livestock and Poultry Product Safety Engineering, Institute of Animal Husbandry and Veterinary Medicine, Anhui Academy of Agricultural Sciences, Hefei 230031, China; xujuanella@163.com (J.X.); lq798711247@163.com (Q.L.); zhanghuibin1997@126.com (H.Z.); zhaoshuanping@163.com (S.Z.); jinhaizjm@163.com (H.J.); lqg3375@163.com (Q.L.); duxinyi2018@163.com (X.D.); 2 College of Animal Science and Technology, Anhui Agricultural University, Hefei 230031, China; jsh3235@126.com

**Keywords:** cattle breeds, meat quality, fatty acids, amino acids, flavor, animal breeding, genetic resources

## Abstract

This study aimed to evaluate breed-specific differences in meat quality, nutritional compo sition, and volatile flavor compounds of beef, and to discuss their implications for animal breeding and health. Longissimus dorsi muscles were collected from four cattle breeds—Dabieshan cattle (DBS), local water buffalo (LWB), Simmental (SM), and Angus (AG)—with six animals per breed, all reared under identical feeding conditions. Meat quality parameters (color, cooking loss, shear force, and water-holding capacity), proximate composition, fatty acid and amino acid profiles, and volatile compounds were analyzed using standardized methods. Significant breed effects were observed for most traits (*p* < 0.05). DBS exhibited the highest intramuscular fat content (2.9%) and total fatty acid concentration, along with favorable proportions of unsaturated fatty acids (MUFA + PUFA) and abundant flavor volatiles (aldehydes and ketones). LWB showed superior water-holding capacity, lowest cooking loss, and the highest total amino acid content, including essential amino acids, along with a higher PUFA/SFA ratio. SM and AG had intermediate meat quality traits, with AG displaying lower lightness (L*) and better tenderness than SM. Notably, LWB and DBS presented higher levels of nutritionally beneficial fatty acids (e.g., C18:3n3) and amino acids (e.g., glutamic acid). Breed significantly influences meat quality and nutritional composition. DBS and LWB offer distinct advantages in fat deposition, amino acid profile, and potential health benefits. These findings provide preliminary insights for selective breeding programs aiming to improve meat quality and meet consumer preferences. The observed compositional differences—such as lower saturated fatty acids in LWB and higher intramuscular fat in DBS—may offer nutritional advantages from a dietary perspective, although direct health benefits were not assessed. The results also underscore the importance of preserving indigenous cattle genetic resources for sustainable animal production.

## Simple Summary

This study compared meat quality and nutritional composition across four cattle breeds—Dabieshan cattle, local water buffalo, Simmental, and Angus—raised under the same feeding conditions. We found that Dabieshan cattle had higher fat content and more abundant flavor compounds, whereas local water buffalo showed better water-holding capacity and higher levels of certain amino acids and a more favorable fatty acid profile from a nutritional standpoint. Simmental and Angus cattle had intermediate meat quality traits. These differences indicate that breed plays an important role in determining meat characteristics, including taste and nutritional value. Understanding these breed-specific traits can help farmers and breeders select cattle that produce healthier and more flavorful meat, while also supporting the conservation of local breeds. This knowledge is valuable for improving animal production systems and meeting consumer demand for high-quality beef.

## 1. Introduction

Beef, prized for its high protein content, abundant minerals, and distinctive flavor, has become a globally esteemed high-value meat [1,2]. According to preliminary estimates by the Food and Agriculture Organization of the United Nations (FAO), global beef production is projected to reach approximately 60 million tons in 2024. The United States, Brazil, and China rank as the top three producers, with their output and trade dynamics significantly influencing the international market landscape [3]. China stands not only as a major producer but also as one of the fastest-growing and their output consumer markets. In recent years, due to rising incomes and dietary upgrades have heightened consumer focus on beef’s sensory qualities and nutritional value, driving demand for premium products rich in protein, low in fat, and abundant in micronutrients [4]. Within beef quality assessment systems, visual attributes like color, texture, and marbling, alongside nutritional properties such as protein and fatty acid composition, collectively form the core criteria for consumer selection. Meat color is primarily determined by myoglobin content and its chemical state, regulated by factors such as breed and feeding conditions [5]. Texture largely depends on muscle water-holding capacity, with juice loss during processing and storage significantly affecting meat firmness and juiciness [6,7]. Additionally, tenderness, flavor, and juiciness are regarded as key indicators of beef palatability, with tenderness being closely related to muscle fiber structure and intramuscular fat content [8].

The variation in beef quality is influenced by multiple factors, including genetic background, sex, age, management practices, and nutritional differences. Numerous studies have demonstrated significant differences among cattle breeds in muscle histological characteristics, fat deposition capacity, and flavor precursor composition. For instance, Angus cattle are renowned for their abundant marbling and superior tenderness [9,10], whereas Simmental cattle are widely promoted for their high meat yield and uniform texture [11]. Water buffalo also exhibit unique meat characteristics, such as higher water-holding capacity and a distinctive fatty acid composition [12]. However, systematic comparative studies on meat quality between indigenous Chinese cattle breeds (e.g., Dabieshan cattle) and imported breeds (e.g., Angus cattle and Simmental cattle), as well as local water buffalo under identical feeding conditions, remain relatively scarce. In particular, comprehensive comparative research on physicochemical properties, fatty acid composition, and flavor compounds is notably insufficient. Therefore, the primary objective of this study is to systematically compare breed-specific differences in meat quality, nutritional composition, and volatile flavor compounds under strictly controlled, identical feeding conditions, thereby addressing this knowledge gap. The Dabieshan cattle are an important local breed in Anhui Province, characterized by their ability to thrive on coarse feed, strong disease resistance, tender meat texture, and rich flavor [13]. Research indicates that this breed possesses a high proportion of essential amino acids (approximately 39.65%) and demonstrates weight gain potential during specific growth stages (e.g., 0–6 months and 12–24 months), suggesting superior beef production potential. In contrast, local water buffalo, as a significant dual-purpose livestock breed for meat and labor, also lack systematic studies of their meat characteristics, particularly a comprehensive exploration of flavor and lipid composition.

In the broader context of livestock production, it is increasingly recognized that animal production systems contribute simultaneously to food security and environmental challenges, and simplistic interpretations of animal production should be avoided [14]. Breed selection, as a key determinant of meat quality and production efficiency, plays an important role in balancing these trade-offs. Consequently, understanding breed-specific meat quality traits is not only relevant for consumer preference but also for sustainable production system design. Accordingly, this study aims to systematically compare the differences in conventional meat quality, physicochemical properties, fatty acid composition, and volatile flavor compounds among Dabieshan cattle, local water buffalo, Simmental cattle, and Angus cattle under identical commercial rearing conditions. The findings are expected to provide theoretical support for the scientific utilization of local yellow cattle germplasm resources and establish a data foundation for breed selection and differentiated processing of high-quality beef, thereby promoting the high-quality development of China’s beef cattle industry.

## 2. Materials and Methods

### 2.1. Animal Ethics Statement

Institute of Animal Science and Veterinary Medicine, Anhui Academy of Agricultural Sciences, approved all animal experiments with permission number IAS2020-48. All the slaughter and sampling procedures strictly complied with the Guidelines on Ethical Treatment of Experimental Animals of China.

### 2.2. Animal and Sample Preparation

The study used 30-month-old adult bulls of four breeds: Anhui DBS (DBS, *n* = 6), Anhui water buffalo (LWB, *n* = 6), SM cattle (SM, *n* = 6), and AG cattle (AG, *n* = 6). All bulls were housed together at a local beef cattle facility in Anhui and fed the same total mixed ration (TMR) for 12 months, formulated with reference to the Feeding Standard of Beef Cattle (NY/T815-2004) [15]. Although a sample size of six per breed is relatively limited, it is consistent with previous comparative meat quality studies on cattle breeds (e.g., Ge [16] and Liu [17]). The sample size was determined by animal availability and experimental feasibility, and the results should be interpreted as preliminary. Future studies with larger cohorts are warranted to validate these findings. The dietary composition and nutritional levels of each experimental group are detailed in Table 1. At the end of the trial, the bulls were fasted for 24 h. After stunning, they were slaughtered following GB/T 19477-2004 [18]. At 24 h post mortem, 1 kg of Longissimus dorsi (LD) was excised between the 12th and 13th ribs. Samples were vacuum-packaged and stored at −80 °C for subsequent analyses.

### 2.3. Meat Quality Measurements

To measure meat quality, we segmented the longissimus dorsi from the carcasses in accordance with the China National Beef Carcass and Cuts Standards (GB/T 27643-2011) [19].

#### 2.3.1. Nutritional Ingredients Determination

The crude protein content of the samples was determined by the Kjeldahl method, in accordance with the China National Determination of Protein in Foods Standards (GB 5009.5-2016) [20]. The formula for calculating the protein content is as follows: $X=\frac{(V_{1}-V_{2})\times c\times 0.0140}{m\times V_{3}/100}\times F\times 10$. Here, *X* represents the protein content of the sample, *V*_1_ is the volume of the test solution of hydrochloric acid standard titration solution, *V*_2_ is the volume of the blank solution of hydrochloric acid standard titration solution, c is the density of the hydrochloric acid standard titration solution, m is the mass of the sample, *V*_3_ is the volume of the digestion liquid, and *F* is the nitrogen–protein conversion factor. The total fat was determined by acid hydrolysis, following the National Standard of China for the determination of fat content in food (GB 5009.6-2016) [21]. The formula for calculating the fat content is: *X* = (*m*_1_ − *m*_0_)/*m*_2_ × 100, where *X* represents the fat content, *m*_1_ is the mass of the fat and Soxhlet extraction flask, *m*_0_ is the mass of the flask, and *m*_2_ is the mass of the sample. The moisture content was determined by the distillation method in the National Standard of China for the determination of moisture content in food (GB 5009.3-2016) [22], and the calculation formula is *X* = (*m*_1_ − *m*_2_)/(*m*_1_ − *m*_3_) × 100, where *X* represents the moisture content, *m*_1_ is the mass of the flask with the sample, *m*_2_ is the mass of the flask with the sample after drying, and *m*_3_ is the mass of the flask.

#### 2.3.2. pH Measurement

The pH of the samples was measured on longissimus dorsi utilizing a TESTO 205 Meat pH meter (TESTO, Shenzhen, China). The mean value was calculated after three measurements of the same part. Prior to use, the pH meter was calibrated with standard solutions (pH 4.0 and 7.0).

#### 2.3.3. Meat Color Measurement

Color measurements, including lightness (L*), redness (a*), and yellowness (b*), were taken three times on each LD muscle sample using the NR10QC portable chromaticity analyzer (3nh Technology Co. Ltd., Shenzhen, China), avoiding fat and connective tissue within the muscle. The average value was calculated as the final meat color value.

#### 2.3.4. Cooking Loss and Warner–Bratzler Shear Force (WBSF) Measurement

To measure the shear force of each sample, 200 g of the sample was selected and cut into blocks measuring 6 cm × 3 cm × 3 cm. The samples were then heated to 70 °C in a water bath, with the core temperature of each specimen continuously monitored using a digital thermometer for 20 min. After cooling the sample to room temperature, it was divided into 1 cm × 1 cm × 3 cm cuboids. Shear force was measured using a V-blade (1 mm thick, 60° internal angle, 40 mm internal notch height) with an initial force of 0.38 N and a test rate of 60 mm/min. Each sample group underwent three parallel measurements, and the average value was calculated as the final shear force value. Specific parameters refer to Ji et al. (2014) [23]. Water-holding capacity was measured using a 25 kg press and a 300 s compression time. Water loss rate was determined by calculating the percentage weight loss: (*M*_1_ − *M*_2_)/*M*_1_ × 100%, where *M*_1_ represents meat weight before compression, and *M*_2_ represents meat weight after compression. The meat water-holding capacity was measured using a meat water-holding capacity tester (MAEC-18). To determine cooking loss, 200 g of each sample was heated to 70 °C in a water bath. Cooking loss rate was calculated as the percentage weight loss during cooking using the formula (*W*_1_ − *W*_2_)/*W*_1_ × 100%, where *W*_1_ and *W*_2_ represent the initial and final meat weights, respectively.

#### 2.3.5. Amino Acid Measurement

In accordance with methods of the China National Determination of Amino Acids in Foods Standards (GB 5009.124-2016) [24], we employed ion exchange chromatography coupled with post-column derivatization of indanedione to analyze 16 distinct amino acid components and their respective content levels. The mixed amino acid standard solution and the sample solution were injected into the amino acids analyzer at the same volume, respectively, and the amino acid concentration in the sample determination solution was calculated by the peak area [25]. The amino acid content of the sample solution was calculated with *c_i_* = *c_s_*/*A_s_* × *A_i_*. Here, *c_i_* represented the content of amino acid *i* in the sample solution, *A_i_* was the peak area of amino acid *i*, *A_s_* was the peak area of amino acid *s* in the amino acid standard solution, and cs was the content of amino acid *s*. Each amino acid content was calculated with *X_i_* = *c_i_* × *F* × *V* × *M*/*m* × 10^9^ × 100, where *X_i_* was the content of amino acid *i*, *F* referred to the dilution ratio, *V* was the volume of the sample hydrolysate, and *M* was the molar mass of amino acid *i*. Each sample was analyzed in duplicate, and the average value was used for calculation.

#### 2.3.6. Fatty Acids Composition

Following the guidelines established in the China National Determination of Fatty Acids in Foods Standards (GB 5009.168-2016) [26], we extracted and analyzed total fatty acids from the samples using a GC-2014 C gas chromatograph detector (Shimadzu Co., Ltd., Kyoto, Japan). We utilized 0.5 g of freeze-dried meat powder from each sample to determine the composition and content of 37 individual fatty acids. The samples were extracted by hydrolyzation-ether solution, and then saponified and methylated under alkaline conditions to produce fatty acid methyl ester. The content of fatty acid methyl ester was quantitatively determined by the internal standard method through capillary column gas chromatography. The specific steps were described in a previous study [27]. The fatty acid content was calculated following the formula *X_Fatty acids_* = *X_FAME_* × *F_FAME−FA_*, where *X_fatty acids_* was the fatty acid content, *X_FAME_* was the methyl ester content, *F_FAME−FA_* was the conversion coefficient of fatty acid methyl ester to fatty acid calculated by *F_FAME−FA_* = *M_FA i_*/*M_FAME I_*, *M_FA i_* was the molecular mass of fatty acid *i*, and *M_FAMEi_* was the molecular mass of methyl ester.

#### 2.3.7. Analysis of Volatile Compounds by GC-HRMS

The method of Yang et al. (2022) [28] was employed to analyze aroma volatiles. In brief, samples were trimmed of fascia and surface fat, ground in a grinder, placed in cooking bags, and heated in an 80 °C water bath for 30 min before removal and cooling to room temperature. A 3 g minced sample was introduced into a 20 mL glass vial. The vials were immediately closed with a magnetic cap fitted with a polytetrafluoroethylene-silicone septum. The sample vial was incubated at 55 °C for 20 min and extracted at 55 °C for 40 min using a 50/30 μm Divinylbenzene/Carboxen/Polydimethylsiloxane (DVB/CAR/PDMS) fiber (Supelco, Inc., Bellefonte, PA, USA). In order to ensure faster extraction, the vial was maintained in agitation during the extraction period. Once the extraction was finished, the fiber was automatically inserted into the injector and desorbed at 250 °C for 3 min. Between the consecutive analyses, the fiber was conditioned in the other injector port at 270 °C for 10 min. A VF-WAX ms column (60 m × 0.25 mm i.d. × 0.25 μm film thickness, Agilent, Santa Clara, CA, USA) was used. Helium (99.9999%) with a constant flow rate of 1 mL/min was used as the carrier gas. The column oven was temperature-programmed starting at 40 °C for 2 min, then increased to 230 °C at a rate of 4 °C/min and then maintained at 230 °C for 5 min. Both the transfer line 1 and transfer line 2 were set at 250 °C. MS was performed using electron impact ionization (EI) at 70 eV, operating in full scan mode at a resolving power of 60,000 full width at half maximum (FWHM). The scan range was from 30 to 400 *m*/*z* with an automatic gain control target value of 1 × 10^6^. Ion source and transfer line temperatures for MS were set at 280 °C and 250 °C, respectively. GC–MS data were acquired and processed using the Xcalibur 4.1 and TraceFinder 4.0 software (Thermo Scientific), respectively. Volatile compounds were identified in accordance with mass spectra and linear retention indices (LRIs) from NIST17 (v2.3) and the domestic library.

### 2.4. Statistical Analysis

Data analysis was conducted using IBM SPSS Statistics for Windows, version 25.0 (IBMCorp., Armonk, NY, USA). To compare meat quality parameters, nutritional composition, fatty acids, amino acids, and volatile compounds among the four cattle breeds, a one-way analysis of variance (ANOVA) was performed. When significant F-values were detected (*p* < 0.05), Tukey’s HSD post hoc test was applied for multiple comparisons. The results are presented as mean ± standard deviation (SD) (*n* = 6 per breed). A significance level of *p* < 0.05 was used to determine statistically significant differences. The raw data of volatile compound abundances were subjected to a log2 transformation to normalize their distribution and reduce the impact of extreme values prior to subsequent statistical analysis.

## 3. Results

### 3.1. Nutritional Ingredients Analysis

The conventional nutritional composition of the four cattle breeds (Table 2) revealed significant differences in moisture and total fat content among breeds (*p* < 0.05), while crude protein content remained relatively stable with no significant inter-breed variation (*p* > 0.05). The intramuscular fat content of DBS (2.9%) was approximately four-fold higher than that of LWB (0.67%), a difference likely to be perceptible to consumers. Consistent with this, the moisture content of DBS (71.27 ± 1.32%) was significantly lower than that of LWB (74.22 ± 0.59%), SM (73.77 ± 1.44%), and AG (73.32 ± 0.70%) (*p* < 0.05). Crude protein content showed no statistically significant differences among the four breeds (*p* > 0.05), with values of 23.22 ± 1.48% for DBS, 23.2 ± 0.83% for LWB, 23.1 ± 0.49% for SM, and 23.22 ± 0.65% for AG. Total fat content showed significant differences, exhibiting a descending trend: DBS > AG > SM > LWB. DBS (2.9 ± 1.2 g/100 g) significantly exceeded AG (1.7 ± 0.43 g/100 g), SM (1.0 ± 0.31 g/100 g), and LWB (0.67 ± 0.38 g/100 g), with LWB exhibiting the lowest total fat content.

### 3.2. Meat Quality Analysis

As shown in Table 3, significant differences (*p* < 0.05) were observed in the meat quality characteristics of the longissimus dorsi muscle from the back among the four cattle breeds. The DBS exhibited the highest cooking loss rate (28.82 ± 2.79%), significantly higher than LWB (20.76 ± 4.80%) and AG (22.37 ± 4.38%), but not significantly different from SM (26.57 ± 4.60%). Conversely, LWB exhibited the lowest cooking loss rate, significantly lower than DBS and SM cattle. Shear force results indicated that DBS possessed superior tenderness characteristics, with a shear force value (3.52 ± 0.56 kgf) significantly lower than those of the other three breeds (*p* < 0.001). AG (4.70 ± 0.77 kgf) and SM (5.13 ± 0.63 kgf) exhibited intermediate tenderness, while LWB (7.10 ± 1.10 kgf) showed the poorest tenderness. Water-holding capacity did not differ significantly among the four breeds (*p* > 0.05). Meat color analysis revealed that AG exhibited the lowest lightness value (L* = 21.69 ± 2.12), significantly lower than that of other breeds (*p* < 0.05). LWB showed the highest redness value (a* = 20.25 ± 1.33), while AG had the lowest yellowness value (b*) (6.17 ± 0.95), significantly lower than that of DBS.

### 3.3. Fatty Acid Composition

As shown in Table 4, significant differences (*p* < 0.05) were observed in the total fatty acid content and composition of the longissimus dorsi muscle among different cattle breeds. The total fatty acid content of DBS (2.493 g/100 g) was nearly four times that of LWB and SM, highlighting a remarkable breed difference in lipid deposition. Regarding fatty acid composition, total saturated fatty acids (SFAs) and unsaturated fatty acids (UFAs) were significantly highest in DBS, followed by AG, while LWB and SM showed significantly the lowest levels. Specifically, the content of key saturated fatty acids (C16:0 and C18:0) and monounsaturated fatty acids (C16:1 and C18:1n9c) was highest in DBS, followed by AG, whereas LWB and SM exhibited the lowest levels. Total polyunsaturated fatty acids (PUFAs) were highest in DBS (0.091 ± 0.013 g/100 g), lowest in SM (0.017 ± 0.011 g/100 g), with LWB and AG being intermediate and showing no significant differences. The linoleic acid content (C18:2, n6c) of DBS was higher than that of the other three breeds (*p* < 0.05). The linolenic acid content (C18:3n3) of LWB and DBS was higher than that of AG (*p* < 0.05), whereas the arachidonic acid (C20:1) content in DBS was higher than that in AG. The arachidonic acid (C20:4, n6) content in DBS was higher than that in both LWB and AG (*p* < 0.05).

### 3.4. Amino Acid Composition

**Figure 1:**
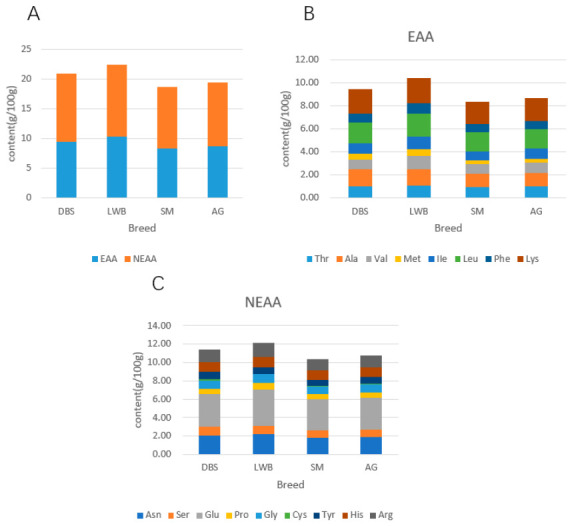
Comparison of amino acid compositions among different beef breeds. (**A**) Comparison of EAAs and NEAAs; (**B**) comparison of EAA compositions; and (**C**) comparison of EAA compositions.

This study detected 17 amino acids in beef from the four breeds. The average total amino acid (TAA) content was 20.39 g/100 g, with essential amino acids (EAAs) and non-essential amino acids (NEAAs) averaging 9.21 g/100 g and 11.18 g/100 g, respectively. As shown in Table 5, significant differences (*p* < 0.05) existed in the amino acid composition of the longissimus dorsi muscle among different cattle breeds. Notably, LWB exhibited the highest total amino acid content, with significantly higher TAA (22.49 ± 1.43 g/100 g), EAA (10.31 ± 0.64 g/100 g), and NEAA (12.17 ± 0.80 g/100 g) contents than other breeds. DBS ranked second in TAA (20.93 ± 1.28 g/100 g), EAA (9.50 ± 0.57 g/100 g), and NEAA (11.43 ± 0.71 g/100 g), significantly exceeding SM and AG. Specific amino acid analysis (Figure 1B,C) revealed that LWB exhibited the highest levels of key essential amino acids, including valine (1.16 ± 0.05 g/100 g), isoleucine (1.10 ± 0.07 g/100 g), leucine (1.96 ± 0.15 g/100 g), lysine (2.19 ± 0.14 g/100 g), and arginine (1.56 ± 0.10 g/100 g). DBS showed superior aspartic acid (2.07 ± 0.15 g/100 g) and glutamic acid (3.55 ± 0.27 g/100 g) levels, as well as threonine, serine, and alanine. SM and AG exhibited significantly lower levels of most amino acids compared to LWB and DBS, though their levels of certain amino acids (e.g., lysine, arginine, valine) were similar. Significant differences in EAA and NEAA content were observed among breeds (Figure 1A). Regarding essential amino acids (EAAs), DBS and LWB exhibited higher EAA levels (9.50 ± 0.57 g/100 g, 10.31 ± 0.64 g/100 g), whereas SM and AG exhibited lower EAA levels (8.38 ± 0.42 g/100 g and 8.65 ± 0.40 g/100 g, respectively).

### 3.5. Volatile Flavor Compounds

Under the current experimental conditions, 84 volatile compounds were detected and quantified in the longissimus dorsi muscle of four breeds (Table 6). Aldehydes (20 compounds) constituted the predominant aroma group, followed by alcohols (10 compounds), ketones (nine compounds), acids (10 compounds), other compounds (11 compounds), esters (eight compounds), and heterocyclic compounds (five compounds). Results indicated that DBS exhibited significantly higher levels of most flavor compounds compared to other breeds (*p* < 0.05). Aldehydes, as the predominant volatile components, showed higher total concentrations in DBS. Specifically, the levels of n-nonanal (15.69 ± 2.58), hexanal (17.28 ± 3.4), and benzaldehyde (18.13 ± 9.46) were significantly higher than those in LWB, SM, and AG. Among alcohols, 1-octen-3-ol (16.95 ± 1.64) and 1-hexanol (15.58 ± 1.75) showed significantly higher levels in DBS (*p* < 0.05), while no significant differences were observed between SM and AG (*p* > 0.05). Ketones such as 2,3-octanedione (18.55 ± 4.43) and 3-octanone (11.47 ± 3.31) were also present at higher concentrations in DBS. Among acids, caproic acid (13.01 ± 0.95) and acetic acid (13.6 ± 4.88) exhibited higher concentrations in DBS. Heterocyclic compounds like 2-pentylfuran (13.62 ± 1.43) and other compounds, such as styrene (10.34 ± 0.63), were also significantly higher in DBS than those in other breeds.

**Figure 2:**
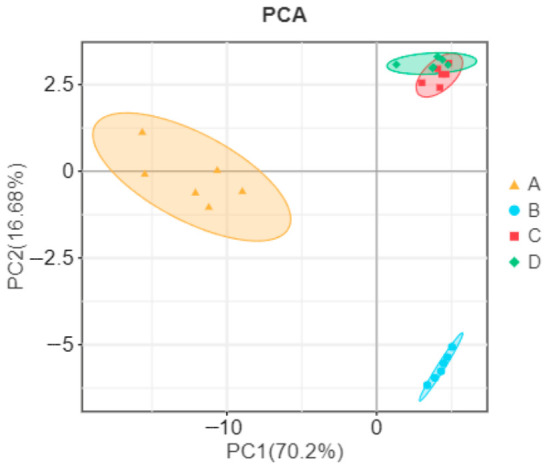
Principal component analysis (PCA) score plot of volatile compounds in longissimus dorsi muscle from four cattle breeds. Each point represents an individual sample (*n* = 6 per breed). PC1 explained 70.2% of the total variance, and PC2 explained 16.88% (cumulative 87.08%). DBS (Dabieshan cattle, ▲) is clearly separated from LWB (local water buffalo, ●), SM (Simmental, ■), and AG (Angus, ◆) along PC1. LWB, SM, and AG partially overlap but show breed-specific tendencies.

To facilitate interpretation of this complex dataset, principal component analysis (PCA) was performed on the 84 volatile compounds. The first two principal components explained 70.2% and 16.88% of the total variance, respectively, cumulatively accounting for 87.08% of the variance. As shown in the PCA score plot (Figure 2), samples from DBS formed a distinct cluster separated from LWB, SM, and AG along PC1. LWB, SM, and AG partially overlapped but still exhibited breed-specific tendencies. This separation indicates that the volatile compound profile of DBS is markedly different from that of the other three breeds, consistent with the higher abundance of aldehydes, ketones, and other flavor compounds observed in DBS (Table 6).

## 4. Discussion

### 4.1. Comparison of Nutritional Components

This study compared the nutritional composition of the longissimus dorsi muscle among DBS, LWB, SM, and AG. Results showed no significant difference in crude protein content among the four breeds (*p* > 0.05), with values around 23%. This finding may be attributed to standardized husbandry practices, such as uniform dietary formulations and environmental conditions, which promote consistent protein deposition. However, the potential influence of genetic factors on protein deposition warrants further investigation. Regarding fat content (Table 2), significant differences were observed among breeds (*p* < 0.05). Among them, DBS exhibited the highest intramuscular fat content, approximately double that of LWB, SM, and AG. This finding corroborates the negative correlation between moisture and fat content in meat [29]: LWB, with the lowest fat content, exhibited the highest moisture content, whereas DBS, due to their highest fat deposition, showed the lowest moisture content. Notably, the moisture content of all four breeds in this study (71.27–74.22%) was significantly higher than that reported for Japanese Black cattle (approximately 45.4%) by Hirai et al. [30]. This substantial difference may stem from breed characteristics or variations in measurement methods. The superior intramuscular fat content of DBS indicated its potential for enhanced culinary quality. Intramuscular fat was closely linked to meat juiciness, tenderness, and flavor development—key attributes that govern beef palatability and consumer preference.

Notably, the relatively large standard deviation for total fat content in DBS (2.9 ± 1.2 g/100 g, CV ≈ 41%) indicates considerable within-breed variation, likely reflecting individual differences in fat deposition capacity despite identical feeding conditions (see Section 4.6). Furthermore, while the negative correlation between moisture and fat content was originally documented in Hanwoo cattle [29], this relationship has been widely observed across mammalian species, supporting the inverse pattern found in the present study (e.g., LWB with the lowest fat and highest moisture, and DBS with the highest fat and lowest moisture).

### 4.2. Comparison of Meat Quality

Meat quality is a complex composite trait that typically requires systematic evaluation through multiple indicators, primarily including sensory and physical attributes such as cooking loss, meat color, water-holding capacity, and shear force [16]. Research indicates significant differences in sensory quality and physicochemical characteristics among beef breeds, primarily attributable to variations in intramuscular fat content and muscle fiber structure [31]. Among these, meat color serves as a crucial indicator for assessing freshness and consumer acceptance, typically quantified using L*, a*, and b* values. Research indicates that lower L* values, higher a* values, and lower b* values generally correspond to higher myoglobin content and a fresher meat appearance [17]. In this study, water buffalo exhibited the optimal muscle color, followed by DBS, indicating these two breeds possess certain advantages in visual appeal.

Water-holding capacity and cooking loss are key parameters affecting meat processing yield and economic value. Among the four breeds examined in this study, water buffalo demonstrated lower cooking loss and higher water-holding capacity, while AG and SM exhibited intermediate values. This difference may be related to the higher water content in buffalo muscle tissue and the specificity of its muscle fiber structure [7]. This result aligns with the water-holding capacity evaluation system established by previous studies, further supporting the negative correlation between water-holding capacity and cooking loss [32].

Regarding the poor tenderness of water buffalo meat (shear force 7.10 kgf, approximately twice that of DBS), this is consistent with previous reports on buffalo meat [12]. The higher shear force may be attributed to larger muscle fiber diameter, higher collagen content, and lower intramuscular fat deposition in buffalo compared to beef breeds. Moreover, the slower post mortem glycolysis rate in buffalo muscle may affect calpain-mediated proteolysis, leading to less tenderization during aging. Further studies on muscle fiber typing and collagen cross-linking are needed to clarify the underlying mechanisms.

Fat content is a crucial factor influencing consumer purchasing behavior, particularly intramuscular fat (IMF) content, which directly affects meat juiciness and flavor [33]. Moisture content often exhibits a negative correlation with fat content, where lower moisture typically indicates higher fat deposition and improved meat palatability. The findings of this study align with those reported by Im et al. (2024) [34], confirming the positive impact of fat on enhancing beef tendern Research indicatesess and highlighting the crucial role of fat deposition in elevating meat-eating quality.

### 4.3. Comparison of Fatty Acid Differences

To contextualize our findings on fatty acid composition, Wood et al. (2008) [35] provided a comprehensive overview of how fatty acid profiles influence meat quality and nutritional value. Consistent with that review, our study shows that breed significantly affects the proportion of saturated and unsaturated fatty acids. In recent years, extensive research has been conducted on the fatty acid composition of beef, with a focus on sensory characteristics, meat flavor, and nutritional value. The fatty acid content in meat serves as a crucial indicator of its nutritional value and is also a key factor determining its cooking flavor [36]. In this study, DBS exhibited the highest total fatty acid content, significantly exceeding that of the other three breeds. This variation may be attributed to breed genetic background, as Wang et al. [37] noted significant differences in fat metabolism and deposition capacity between Simmental cattle and Hanxuan Yellow cattle. In this study, C16:0 (palmitic acid) and C18:0 (stearic acid) were the predominant saturated fatty acids in DBS and AG, while LWB and SM exhibited lower levels of these fatty acids (Figure 1B). Previous studies have indicated that certain SFAs commonly found in meat, particularly C16:0 and C18:0, elevate total cholesterol and low-density lipoprotein levels, constituting risk factors for coronary heart disease [38]. Therefore, the lower C16:0 and C18:0 levels in LWB and SM may align more closely with dietary recommendations that limit saturated fat intake, although the direct health impact would depend on overall diet and consumption patterns. Similarly, studies by Briggs et al. (2017) [39] and Pighin et al. (2016) [40] indicated that SFAs were also key contributors to cardiovascular disease development and were associated with cancer, obesity, diabetes, and other health issues. Dietary recommendations advocate for low-saturated fat foods. LWB and SM cattle may exhibit superior SFA profiles compared to DBS and AG, featuring significantly reduced C16:0 and C18:0 levels, along with a tendency toward lower C14:0 and total SFA content.

Saturated fatty acids (SFAs), monounsaturated fatty acids (MUFAs), and polyunsaturated fatty acids (PUFAs) constitute the primary fatty acid components in meat products. Existing research indicates that increasing unsaturated fatty acid (UFA) intake offers health benefits, particularly in reducing the risk of cardiovascular disease [28]. Oleic acid (C18:1n9c), a key monounsaturated fatty acid, has been extensively documented to offer potential health benefits, which have been associated with favorable lipid profiles in nutritional studies [41]. In this study, DBS and AG exhibited higher oleic acid content, primarily accounting for their significantly greater UFA levels compared to the other two breeds. This suggests DBS may possess unique advantages in fatty acid composition. MUFA constituted the largest proportion of UFAs, primarily composed of oleic acid. The positive health effects of oleic acid have been validated in multiple studies, such as its ability to lower low-density lipoprotein cholesterol (LDL-C) without affecting high-density lipoprotein cholesterol (HDL-C) levels, thereby preventing atherosclerosis [42]. Furthermore, oleic acid content was closely linked to fat texture; a higher proportion of C18:1n9c enhanced fat softness and improved beef flavor and overall palatability [43]. The present findings, which indicated significant variation in fatty acid composition and oleic acid content among breeds, aligned with conclusions from studies on other cattle populations reported by Liu et al. (2020) [17] and Domingo et al. (2015) [41]. These results further supported the notion that breed is a key factor influencing beef fatty acid composition.

Furthermore, C18:3n3 is a polyunsaturated fatty acid (PUFA) recognized for its health benefits in humans [44]. PUFAs have been associated with reduced cardiovascular risk in epidemiological studies and may delay the onset of atherosclerotic conditions [45]. Consequently, ongoing efforts aim to enhance the UFA profile in beef to deliver more desirable products that meet consumer demands. In our study, DBS exhibited significantly higher PUFA content than other breeds, particularly notable for elevated levels of C18:2, n6c (linoleic acid), and C18:3, n3 (α-linolenic acid). Linoleic acid and α-linolenic acid are essential fatty acids for humans, possessing important physiological functions [46]. In summary, buffalo meat may be more suitable for the dietary needs of individuals at high risk for cardiovascular disease due to its lower SFA content. DBS and AG exhibit certain advantages in UFA content, particularly in health-beneficial oleic acid and essential fatty acids, making them ideal choices that balance nutritional value and sensory quality. Future research should further investigate the specific impacts of these fatty acid composition differences on actual cooking quality and health effects to better guide production and consumption.

### 4.4. Comparative Analysis of Amino Acid Composition

Amino acids are the fundamental building blocks of proteins and are essential for all biological processes in living organisms. Beef is considered an ideal animal protein, rich in amino acids such as arginine, glycine, and methionine, which have been demonstrated to play important physiological roles in optimizing human health and development [47]. According to FAO/WHO standards, amino acid composition is considered high quality when EAA/TAA and EAA/NEAA ratios in beef reach 0.4 and 0.6, respectively [16]. Consistent with these criteria, Oh et al. (2016) [48] reported that Hanwoo beef also exhibits favorable amino acid profiles with high essential amino acid ratios and antioxidant activities. The results of this study indicate amino acid composition ratios of 0.45–0.46 for EAA/TAA and 0.81–0.85 for EAA/NEAA, suggesting the selected cattle breeds exhibit favorable amino acid profiles.

Based on similarity in sensory qualities, amino acids are grouped into distinct taste categories: umami (Glu, Asp, Asn, and Gln), sweet (Gly, Ala, Ser, Pro, and Thr), and bitter (Lys, Val, Leu, Ile, Arg, Phe, and Tyr) [49]. Regarding essential amino acids (EAAs), lysine (Lys) and leucine (Leu) were higher in LWB and DBS, while SM and AG exhibited lower levels of these amino acids (Figure 1B). Similarly, DBS and LWB exhibited higher NEAA levels than other breeds. Notably, glutamic acid (Glu) and aspartic acid (Asp)—key amino acids for flavor (umami)—constituted the major components of total amino acids. This finding aligns with Cabezas et al. (2023) [50], who noted that beef with high glutamic acid content exhibits superior flavor and texture. Amino acid ratios are crucial indicators for evaluating beef nutritional quality [51], and the EAA/TAA and EAA/NEAA ratios also varied across breeds. Water buffalo exhibited the highest EAA/TAA ratio, indicating a relatively higher proportion of essential amino acids relative to total amino acids, which may confer nutritional advantages on its meat. In contrast, DBS showed a higher EAA/NEAA ratio, suggesting a relatively higher proportion of essential amino acids relative to non-essential amino acids, potentially linked to higher protein content in their feed.

In summary, significant amino acid composition differences exist among beef breeds, potentially influencing nutritional value and flavor characteristics. DBS and LWB exhibit distinct advantages in essential and non-essential amino acids, while water buffalo demonstrates superior glutamic acid content. These results provide a fundamental basis for consumers to choose different varieties of beef, and also offer references for the quality improvement and nutritional regulation of beef.

### 4.5. Comparative Analysis of Volatile Aroma Compounds

The biochemical origin of volatile compounds, particularly aldehydes and ketones, lies in lipid oxidation and the Maillard reaction [52]. Aroma, as part of flavor perceived by olfactory receptors, is a key determinant of cooked meat sensory quality [53]. During cooking, the oxidation of fatty acids, the Maillard reaction between amino acids and sugars, and interactions among their intermediate products constitute the primary pathways for volatile compound formation [54]. Aldehydes are widely recognized as major contributors to meat aroma due to their low odor detection thresholds, primarily formed through thermal oxidation of fatty acids, with a minor portion potentially originating from Strecker degradation of amino acids [55]. Furthermore, the quality and quantity of aldehydes are considered important indicators for distinguishing species-specific aroma profiles [56]. All four beef types in this study exhibited favorable flavor compound profiles, with DBS showing the most pronounced characteristics, followed by SM. Further analysis revealed that most unsaturated aldehydes detected in the four beef types—including valeraldehyde, nonanal, *E*-2-nonenal, and decanal—were oleic acid derivatives possessing pleasant aromas (e.g., fatty aromas). These compounds significantly contribute to beef flavor due to their low odor thresholds. This phenomenon may be related to the unique rumen microbial community structure of ruminants, which produces specific unsaturated fatty acid precursors through biohydrogenation [57], subsequently forming distinctive flavor compounds during cooking.

Alcohols are flavor compounds generated from polyunsaturated fatty acids—acting as flavor precursors—via lactose fermentation, amino acid metabolism, or aldehyde reduction [58]. Most alcohols possess pleasant odors such as sweet, fresh, fruity, vegetal, and floral notes, which enhance the volatile flavors of meat products. Due to their relatively high odor thresholds, alcohols generally exert minimal influence on the volatile flavor profile of beef, with only a few high-concentration alcohols showing discernible effects. Research indicates that while alcohols contribute less significantly to volatile flavor formation than aldehydes, they exhibit synergistic effects on the overall volatile flavor profile of meat [59].

Ketone compounds such as 2,3-octanedione and 3-octanone primarily impart sweet or fruity notes, aiding in meat flavor development. This aligns with findings by Li et al. (2024) [60] on yak beef, which positively contributes to beef aroma. DBS exhibited significant advantages in key flavor compounds like 2,3-octanedione and 3-octanone. These ketones primarily originate from the Maillard reaction and fatty acid oxidation, exhibiting characteristic sweet and buttery aroma profiles [61]. Of particular note is the relatively high content of 1-octen-3-one detected in DBS. While this compound may impart metallic flavors, it enhances flavor complexity and layering at moderate levels.

Acidic flavor compounds serve as precursors to methyl ketones, alcohols, lactones, and esters [62]. In this study, six straight-chain fatty acids (acetic, butyric, caproic, caprylic, capric, and decanoic acids) and branched-chain fatty acids (3-methylbutanoic, 2-ethylhexanoic, and 2-methylpropanoic acids) were detected. Previous research indicates these acids are major flavor components in many cheese types, contributing to the aroma of highly mature cheeses. Esters are formed through esterification during beef cooking or via microbial activity. Esters with ≤10 carbon atoms typically exhibit fruity aromas, while long-chain esters contribute fatty aromas [63]. In this study, methyl butyrate content was elevated in DBS, with this compound contributing to the fruity characteristics of cooked beef. Analysis based on aroma compounds indicates a significant influence of breed on the volatile components of cooked beef aroma, particularly fat-derived compounds. Beyond flavor chemistry, from a sustainability perspective, breed selection for specific meat quality traits should consider trade-offs with feed efficiency and environmental impact [14].

### 4.6. Limitations

This study has several limitations. First, the sample size of six animals per breed is relatively small, which may limit statistical power and generalizability. Second, animals were sourced from a single commercial facility, so results may not fully represent breed performance under different management or environmental conditions. Third, within-breed variability could not be adequately captured. Fourth, the study did not include actual consumer sensory evaluation or clinical health outcomes; therefore, references to “health benefits” are limited to compositional attributes. Fifth, volatile compound profiles were not directly validated by sensory panels, so inferences about flavor perception remain indirect. Future studies with larger cohorts, multiple farms, and longitudinal designs are needed to validate and extend these findings.

## 5. Conclusions

This study systematically compared the meat quality characteristics of DBS, LWB, SM, and AG. Results revealed significant differences among breeds in nutritional composition, sensory properties, and flavor characteristics. Specifically, DBS had the highest intramuscular fat (2.9%) and total fatty acids (2.49 g/100 g), whereas LWB showed the lowest cooking loss (20.8%) and highest total amino acids (22.5 g/100 g). DBS exhibited superior performance in fat content, meat color, and volatile flavor compounds. Although water buffalo exhibited relatively lower tenderness, it demonstrated excellent water-holding capacity, as evidenced by lower cooking loss rate and higher moisture content. Its lower saturated fatty acid content may appeal to consumers seeking reduced saturated fat intake. Regarding amino acid composition, both water buffalo and DBS performed well, achieving optimal levels of total amino acids and essential amino acid ratios.

From a practical perspective, the distinct traits observed—for example, higher fat content and richer volatile compounds in DBS, and lower saturated fatty acids with higher amino acids in LWB—suggest potential for differentiated market positioning. DBS may be particularly suitable for beef products where marbling and flavor are prioritized, while LWB could be explored for leaner, protein-rich offerings. However, these suggestions are preliminary and require validation under different production systems and with larger sample sizes. Future research should also investigate the genetic mechanisms underlying fat deposition and flavor formation, as well as evaluate how feeding strategies or post-mortem processing can optimize breed-specific advantages. In summary, this study clarifies distinct meat quality traits among cattle breeds, providing scientific references for consumer differentiation and valuable insights for beef cattle breeding programs. Advancing the high-quality development of the beef industry will require further research on the impact of diverse feeding management practices on meat quality.

## 6. Perspectives

This study provides a comparative analysis of meat quality and nutritional traits among four cattle breeds under standardized feeding conditions. Consequently, further investigations are warranted to validate the consistency of these findings across different management systems, geographic regions, and larger sample populations. Future research should explore the underlying genetic mechanisms governing breed-specific differences in fat deposition, fatty acid composition, and amino acid profiles, particularly through transcriptomic and genomic approaches. Furthermore, longitudinal studies assessing the relationship between meat quality traits and animal health indicators—such as immune function, oxidative stress status, and metabolic efficiency—would elucidate the physiological basis of these breed differences. Evaluating the performance of these breeds under varying nutritional or environmental conditions may also contribute to optimizing feeding strategies and improving animal welfare. Taken together, integrating meat quality assessment into routine breeding and health management programs could support the development of more resilient, productive, and consumer-oriented cattle production systems.

## Figures and Tables

**Table 1:** The diet composition of four breeds of cattle in the fattening stage.

Feedstuff (%)	Proportion
Corn	60
Wheat bran	15
Soybean	7
Cottonseed Meal	13
Limestone meal	2
Salt	1
Baking Soda	1
Premix ^1^	1
Whole corn silage	62.5
Straw	37.5
Nutritional Level	
CP (%)	12.6
ME (%)	2.67
Ca (%)	0.69
P (%)	0.34
NDF (%)	34.3
ADF (%)	22.5

^1^ The premix provided per kg of diet: Vit A 10,000 IU; Vit D 2500 IU; Vit E 50 IU; Cu 10 mg; Zn 40 mg; Fe 50 mg; Mn 40 mg; I 0.5 mg; Se 0.3 mg; Co 0.1 mg. CP, crude protein; ME, metabolizable energy; Ca, calcium; P, phosphorus; NDF, neutral detergent fiber; and ADF, acid detergent fiber.

**Table 2:** Comparison of differences in nutritional ingredients.

Composition	Cattle Breed				*p* Value
	DBS	LWB	SM	AG	
Moisture/%	71.27 ± 1.32 ^a^	74.22 ± 0.59 ^b^	73.77 ± 1.44 ^b^	73.32 ± 0.70 ^b^	0.001
Crude protein/%	23.22 ± 1.48	23.2 ± 0.83	23.1 ± 0.49	23.22 ± 0.65	0.994
Total fat g/100 g	2.9 ± 1.2 ^a^	0.67 ± 0.38 ^b^	1.0 ± 0.31 ^c^	1.7 ± 0.43 ^d^	0.006

Values are presented as mean ± SD (*n* = 6). ^a–d^ Means within a row with different superscripts differ significantly (*p* < 0.05). DBS: Dabieshan cattle; LWB: local water buffalo; SM: Simmental; AG: Angus.

**Table 3:** Meat quality properties of longissimus lumborum muscle, categorized by cattle breed.

Items	Cattle Breed				*p* Value
	DBS	LWB	SM	AG	
Cooking loss/%	28.82 ± 2.79 ^a^	20.76 ± 4.80 ^c^	26.57 ± 4.60 ^ab^	22.37 ± 4.38 ^b^	0.013
Shear force/kgf	3.52 ± 0.56 ^c^	7.10 ± 1.10 ^a^	5.13 ± 0.63 ^b^	4.70 ± 0.77 ^b^	0.000
Water-holding capacity/%	25.73 ± 4.55	26.00 ± 2.00	24.03 ± 3.57	22.64 ± 2.81	0.358
CIE L*	34.48 ± 3.41 ^a^	29.51 ± 4.61 ^b^	23.98 ± 2.74 ^c^	21.69 ± 2.12 ^c^	0.000
CIE a*	19.59 ± 1.62 ^a^	20.25 ± 1.33 ^a^	7.22 ± 0.80 ^b^	7.13 ± 1.15 ^b^	0.000
CIE b*	10.11 ± 1.17 ^a^	9.57 ± 0.97 ^a^	10.68 ± 4.65 ^a^	6.17 ± 0.95 ^b^	0.023

Values are presented as mean ± SD (*n* = 6). ^a–c^ Means within a row with different superscripts differ significantly (*p* < 0.05). CIE L* (lightness), a* (redness), and b* (yellowness). DBS: Dabieshan cattle; LWB: local water buffalo; SM: Simmental; and AG: Angus.

**Table 4:** Relative fatty acid content of longissimus dorsi muscle, categorized by beef breed.

Items	Cattle Breed				*p* Value
	DBS	LWB	SM	AG	
Total fatty acids, g/100 g	2.493 ± 0.124 ^a^	0.667 ± 0.378 ^b^	0.604 ± 0.348 ^b^	1.310 ± 0.439 ^c^	0.080
C14:0	0.088 ± 0.004	0.010 ± 0.003	0.021 ± 0.01	0.081 ± 0.102	0.134
C15:0	0.010 ± 0.001 ^a^	-	0.001 ± 0.002 ^b^	0.004 ± 0.007 ^b^	0.003
C16:0	0.763 ± 0.035 ^a^	0.072 ± 0.055 ^b^	0.145 ± 0.073 ^b^	0.643 ± 0.809 ^a^	0.016
C18:0	0.453 ± 0.023 ^a^	0.073 ± 0.065 ^b^	0.131 ± 0.092 ^b^	0.440 ± 0.497 ^a^	0.029
C14:1	0.014 ± 0.001	-	-	0.012 ± 0.017	0.048
C16:1	0.073 ± 0.004 ^a^	0.006 ± 0.003 ^b^	0.01 ± 0.006 ^b^	0.058 ± 0.078 ^a^	0.054
C18:1, n9c	0.978 ± 0.048 ^a^	0.118 ± 0.102 ^b^	0.278 ± 0.160 ^b^	0.648 ± 0.199 ^c^	0.083
C18:2, n6c	0.079 ± 0.010 ^a^	0.028 ± 0.009 ^bc^	0.015 ± 0.009 ^b^	0.035 ± 0.026 ^c^	0.000
C18:3, n3	0.006 ± 0.001 ^a^	0.007 ± 0.002 ^a^	-	0.001 ± 0.002 ^b^	0.000
C20:1	0.004 ± 0.000	-	-	0.002 ± 0.005	0.051
C20:4, n6	0.004 ± 0.001 ^a^	0.012 ± 0.002 ^b^	0.002 ± 0.002 ^a^	0.006 ± 0.004 ^c^	0.000
SFA	1.313 ± 0.061 ^a^	0.155 ± 0.125 ^b^	0.298 ± 0.173 ^b^	0.654 ± 0.236 ^bc^	0.020
UFA	1.156 ± 0.064 ^a^	0.166 ± 0.118 ^b^	0.305 ± 0.174 ^b^	0.610 ± 0.252 ^c^	0.076
MUFA	1.065 ± 0.052 ^a^	0.121 ± 0.106 ^b^	0.288 ± 0.165 ^b^	0.994 ± 0.480 ^c^	0.078
PUFA	0.091 ± 0.013 ^a^	0.045 ± 0.014 ^b^	0.017 ± 0.011 ^c^	0.044 ± 0.035 ^b^	0.000
MUFA/SFA	0.811 ± 0.006	0.792 ± 0.088	0.958 ± 0.115	0.994 ± 0.172	0.000
PUFA/SFA	0.069 ± 0.007 ^a^	0.304 ± 0.25 ^b^	0.058 ± 0.016 ^c^	0.050 ± 0.027 ^c^	0.000

Values are presented as mean ± SD (*n* = 6). ^a–c^ Means within a row with different superscripts differ significantly (*p* < 0.05). SFAs: saturated fatty acids; UFAs: unsaturated fatty acids; MUFAs: monounsaturated fatty acids; and PUFAs: polyunsaturated fatty acids. DBS: Dabieshan cattle; LWB: local water buffalo; SM: Simmental; and AG: Angus.

**Table 5:** Amino acid content (mg/100 g) of longissimus dorsi muscle, categorized by cattle breed.

Items	Cattle Breed				*p* Value
	DBS	LWB	SM	AG	
Aspartic acid	2.07 ± 0.15 ^a^	2.22 ± 0.15^a^	1.80 ± 0.19 ^b^	1.88 ± 0.14 ^b^	0.001
Threonine	1.04 ± 0.07 ^a^	1.07 ± 0.04 ^a^	0.90 ± 0.05 ^b^	0.95 ± 0.05 ^bc^	0.000
Serine	0.92 ± 0.05 ^a^	0.92 ± 0.07 ^a^	0.80 ± 0.05 ^b^	0.84 ± 0.05 ^bc^	0.009
Glutamic acid	3.55 ± 0.27 ^a^	3.87 ± 0.23 ^b^	3.40 ± 0.22 ^a^	3.45 ± 0.27 ^a^	0.012
Proline	0.64 ± 0.04 ^a^	0.74 ± 0.04 ^b^	0.60 ± 0.04 ^c^	0.61 ± 0.02 ^ac^	0.000
Glycine	0.88 ± 0.04 ^a^	0.96 ± 0.07 ^b^	0.80 ± 0.03 ^c^	0.83 ± 0.04 ^ac^	0.000
Alanine	1.43 ± 0.07 ^a^	1.39 ± 0.11 ^a^	1.20 ± 0.05 ^b^	1.21 ± 0.08 ^bc^	0.000
Cysteine	0.17 ± 0.03	-	0.10 ± 0.02	0.12 ± 0.04	0.005
Valine	0.88 ± 0.05 ^a^	1.16 ± 0.05 ^b^	0.80 ± 0.04 ^c^	0.86 ± 0.05 ^ac^	0.000
Methionine	0.48 ± 0.10 ^ac^	0.59 ± 0.07 ^a^	0.30 ± 0.17 ^b^	0.33 ± 0.15 ^bc^	0.003
Isoleucine	0.91 ± 0.06 ^a^	1.10 ± 0.07 ^b^	0.80 ± 0.04 ^c^	0.86 ± 0.03 ^c^	0.000
Leucine	1.82 ± 0.12 ^a^	1.96 ± 0.15 ^b^	1.70 ± 0.06 ^b^	1.71 ± 0.06 ^bc^	0.000
Tyrosine	0.78 ± 0.05 ^a^	0.80 ± 0.06 ^a^	0.70 ± 0.03 ^b^	0.67 ± 0.05 ^bc^	0.000
Phenylalanine	0.79 ± 0.05 ^a^	0.85 ± 0.07 ^a^	0.70 ± 0.03 ^b^	0.70 ± 0.04 ^bc^	0.000
Histidine	1.02 ± 0.04	1.11 ± 0.11	1.00 ± 0.07	1.10 ± 0.06	0.117
Lysine	2.14 ± 0.13 ^a^	2.19 ± 0.14 ^a^	2.00 ± 0.08 ^c^	2.05 ± 0.04 ^ab^	0.007
Arginine	1.42 ± 0.10 ^a^	1.56 ± 0.10 ^b^	1.20 ± 0.05 ^c^	1.25 ± 0.03 ^c^	0.000
EAA	9.50 ± 0.57 ^a^	10.31 ± 0.64 ^b^	8.38 ± 0.42 ^c^	8.65 ± 0.40 ^c^	0.000
NEAA	11.43 ± 0.71 ^ac^	12.17 ± 0.80 ^a^	10.36 ± 0.50 ^b^	10.74 ± 0.49 ^bc^	0.000
TAA	20.93 ± 1.28 ^a^	22.49 ± 1.43 ^b^	18.74 ± 0.91 ^c^	19.4 ± 0.88 ^c^	0.000
EAA/TAA	0.45 ± 0.00 ^a^	0.46 ± 0.00 ^b^	0.45 ± 0.004 ^c^	0.45 ± 0.00 ^c^	0.000
EAA/NEAA	0.83 ± 0.01 ^a^	0.85 ± 0.01 ^b^	0.81 ± 0.01 ^c^	0.81 ± 0.01 ^c^	0.000

Values are presented as mean ± SD (*n* = 6). ^a–c^ Means within a row with different superscripts differ significantly (*p* < 0.05). EAAs: essential amino acids; NEAAs: non-essential amino acids; and TAA: total amino acids. DBS: Dabieshan cattle; LWB: local water buffalo; SM: Simmental; and AG: Angus.

**Table 6:** Comparison of volatile compound components in cattle of different breeds.

Items	Cattle Breed				*p* Value
	DBS	LWB	SM	AG	
Benzenemethanol	8.51 ± 1.48 ^a^	3.09 ± 0.21 ^b^	1.90 ± 0.21 ^c^	2.23 ± 0.39 ^bc^	0.000
1-Hexanol	15.58 ± 1.75 ^a^	2.79 ± 0.53 ^b^	4.21 ± 0.83 ^c^	4.34 ± 1.05 ^c^	0.000
1-Dodecanol	7.41 ± 2.89 ^a^	2.93 ± 0.42 ^b^	1.27 ± 0.42 ^bc^	1.03 ± 0.19 ^c^	0.000
E,6-Nonen-1-ol	5.75 ± 2.37 ^a^	-	1.02 ± 0.24 ^b^	0.81 ± 0.32 ^b^	0.000
3-(Methylthio)-1-propanol	8.70 ± 2.13 ^a^	-	0.10 ± 0.16 ^b^	0.05 ± 0.05 ^b^	0.000
1-Octen-3-ol	16.95 ± 1.64 ^a^	5.88 ± 0.81 ^b^	5.54 ± 0.49 ^b^	5.98 ± 1.50 ^b^	0.000
Z,5-Octen-1-ol	8.01 ± 1.29	4.06 ± 0.71	-	-	0.025
Isopropyl Alcohol	7.25 ± 2.05	-	0.39 ± 0.08	0.42 ± 0.07	0.000
1-Butanol	6.10 ± 1.43	1.55 ± 0.31	-	-	0.045
1-Pentanol	11.57 ± 2.4	-	3.42 ± 0.55	3.90 ± 1.48	0.000
Benzaldehyde	18.13 ± 9.46 ^a^	10.91 ± 0.18 ^b^	9.77 ± 0.22 ^b^	9.89 ± 0.52 ^b^	0.017
Pentanal	10.40 ± 3.16 ^a^	5.75 ± 0.61 ^b^	5.49 ± 0.54 ^b^	5.92 ± 1.57 ^b^	0.000
Heptanal	10.04 ± 2.15 ^a^	7.20 ± 1.13 ^b^	3.71 ± 0.48 ^c^	3.92 ± 0.90 ^c^	0.000
Decanal	10.37 ± 1.63 ^a^	5.32 ± 0.50 ^b^	3.62 ± 0.29 ^c^	3.60 ± 0.30 ^c^	0.000
Dodecanal	10.90 ± 1.55 ^a^	2.72 ± 0.69 ^b^	2.74 ± 0.31 ^b^	2.44 ± 0.30 ^b^	0.000
Benzenacetaldehyde	12.04 ± 0.67	-	4.37 ± 0.35	4.41 ± 0.47	0.000
Nonanal	15.69 ± 2.58 ^a^	10.36 ± 0.58 ^b^	7.89 ± 0.37 ^c^	7.99 ± 0.63 ^c^	0.000
5-Methyl-2-thiophenecarboxaldehyde	6.70 ± 2.71	-	1.67 ± 1.34	2.04 ± 1.26	0.000
E,2-Heptenal	12.94 ± 3.15 ^a^	0.68 ± 0.31 ^b^	2.36 ± 0.51 ^c^	2.89 ± 1.16 ^c^	0.000
E,2-Nonenal	9.06 ± 1.11 ^a^	2.55 ± 0.65 ^b^	2.98 ± 0.61 ^b^	3.43 ± 0.86 ^b^	0.000
5-Ethyl-2-furaldehyde	5.81 ± 1.50	-	0.12 ± 0.02	0.13 ± 0.05	0.000
E,E-2,4-Decadienal	9.14 ± 2.55	-	2.91 ± 0.57	3.47 ± 1.07	0.000
E,2-Octenal	12.77 ± 1.99 ^a^	1.60 ± 0.5 ^b^	3.00 ± 0.51 ^bc^	3.56 ± 1.15 ^c^	0.000
E,2-Decenal	8.76 ± 0.57 ^a^	1.34 ± 0.55 ^b^	2.45 ± 0.56 ^c^	2.71 ± 0.61 ^c^	0.000
Benzaldehyde, 4-ethyl-	11.09 ± 1.05 ^a^	4.03 ± 1.19 ^b^	2.04 ± 0.05 ^c^	2.27 ± 0.36 ^c^	0.000
E,2-Undecenal	10.29 ± 0.64 ^a^	1.07 ± 0.68 ^b^	2.15 ± 0.53 ^c^	2.46 ± 0.56 ^c^	0.000
Butanal, 3-methyl-	7.73 ± 1.59 ^a^	1.25 ± 0.16 ^b^	3.09 ± 0.54 ^c^	3.11 ± 0.56 ^c^	0.000
E,E-2,4-Nonadienal	7.84 ± 1.91	-	2.18 ± 0.49	2.55 ± 0.89	0.000
Hexanal	17.28 ± 3.4 ^a^	9.69 ± 0.68 ^b^	8.85 ± 0.60 ^b^	9.22 ± 1.57 ^b^	0.000
Benzaldehyde, 4-pentyl-	9.55 ± 3.17	-	1.94 ± 0.46	1.79 ± 0.44	0.000
Benzeneacetic acid, methyl ester	8.14 ± 1.49	-	3.13 ± 1.62	2.75 ± 1.62	0.000
Phenethyl acetate	6.62 ± 1.24	-	0.11 ± 0.03	0.41 ± 0.47	0.000
γ-Nonanolactone	5.80 ± 3.09	-	0.34 ± 0.17	0.17 ± 0.04	0.000
Decanoic acid, methyl ester	9.11 ± 3.1 ^a^	0.30 ± 0.26 ^b^	2.90 ± 0.55 ^c^	2.86 ± 0.21 ^c^	0.000
Octyl formate	5.85 ± 2.94	4.97 ± 0.60	3.42 ± 0.87	3.45 ± 1.05	0.326
Methyl 3-hydroxybutyrate	2.98 ± 1.52	-	0.22 ± 0.17	0.32 ± 0.40	0.000
Methyl nonanoate	7.84 ± 5.14	-	3.53 ± 1.23	3.17 ± 0.43	0.279
Butanoic acid, methyl ester	10.74 ± 4.67	-	2.85 ± 0.76	2.59 ± 1.13	0.000
3-Octanone	11.47 ± 3.31 ^a^	1.93 ± 0.46 ^b^	1.39 ± 0.28 ^b^	1.42 ± 0.57 ^b^	0.000
2-Heptanone	9.95 ± 0.97 ^a^	2.97 ± 0.53 ^b^	3.10 ± 0.44 ^b^	2.72 ± 1.00 ^b^	0.000
6-Methyl-5-hepten-2-one	9.24 ± 1.14 ^a^	4.47 ± 0.31 ^b^	2.86 ± 0.13 ^c^	3.17 ± 0.40 ^c^	0.000
2-Acetylpyridine	10.50 ± 1.21 ^a^	4.09 ± 1.89 ^b^	0.20 ± 0.23 ^c^	0.54 ± 0.27 ^c^	0.000
3-Octen-2-one	7.71 ± 2.28	0.24 ± 0.06	-	-	0.000
1-Octen-3-one	8.90 ± 1.46	-	0.95 ± 0.29	1.29 ± 0.74	0.000
Acetoin	6.54 ± 1.69	-	6.17 ± 0.65	6.16 ± 0.71	0.799
2,3-Octanedione	18.55 ± 4.43 ^a^	6.43 ± 1.15 ^b^	6.01 ± 0.84 ^b^	6.61 ± 2.05 ^b^	0.000
Camphor	7.43 ± 1.75	-	1.40 ± 0.08	1.48 ± 0.13	0.000
2-Heptanone, 6-methyl-	11.51 ± 1.47 ^a^	2.04 ± 0.63 ^b^	1.09 ± 0.41 ^b^	1.01 ± 0.46 ^b^	0.000
Butanoic acid	9.43 ± 2.13 ^a^	2.20 ± 0.74 ^b^	1.65 ± 0.63 ^b^	1.67 ± 1.00 ^b^	0.000
Heptanoic acid	9.58 ± 1.47 ^a^	2.86 ± 0.79 ^b^	1.32 ± 0.33 ^c^	0.98 ± 0.34 ^c^	0.000
Octanoic acid	10.27 ± 1.56 ^a^	3.44 ± 0.76 ^b^	2.23 ± 0.39 ^c^	2.00 ± 0.31 ^c^	0.000
Hexanoic acid	13.01 ± 0.95 ^a^	4.12 ± 0.70 ^b^	3.07 ± 0.49 ^c^	3.19 ± 0.97 ^c^	0.000
2-Ethylhexanoic acid	6.53 ± 1.88	3.24 ± 1.26	-	-	0.005
Decanoic acid	8.54 ± 1.99 ^a^	3.10 ± 0.89 ^b^	1.65 ± 0.32 ^c^	1.53 ± 0.37 ^c^	0.000
Butanoic acid, 3-methyl-	8.26 ± 3.05	-	2.46 ± 2.97	1.76 ± 2.90	0.003
Acetic acid	13.60 ± 4.88 ^a^	3.74 ± 1.96 ^b^	3.36 ± 1.17 ^b^	2.96 ± 1.18 ^b^	0.000
Benzoic acid	9.49 ± 1.77 ^a^	2.94 ± 0.65 ^b^	3.17 ± 0.20 ^b^	3.23 ± 0.44 ^b^	0.000
2-Methylpropanoic acid	2.37 ± 1.96	-	1.81 ± 1.87	1.53 ± 1.93	0.748
Benzene, (1-butylheptyl)-	8.63 ± 2.59	-	0.07 ± 0.03	0.04 ± 0.01	0.000
hexadecanal	13.36 ± 2.26	-	7.04 ± 0.43	6.30 ± 0.78	0.000
Dimethyl sulfone	6.44 ± 1.74	-	0.29 ± 0.13	0.26 ± 0.12	0.000
2-Ethylfuran	9.04 ± 2.20 ^a^	3.03 ± 0.84 ^b^	1.58 ± 0.41 ^b^	1.87 ± 0.71 ^b^	0.000
2-Pentylfuran	13.62 ± 1.43 ^a^	6.75 ± 1.20 ^b^	6.77 ± 0.44 ^b^	7.08 ± 1.02 ^b^	0.000
2-Hexylfuran	5.70 ± 1.88 ^a^	0.37 ± 0.24 ^b^	0.15 ± 0.11 ^b^	0.20 ± 0.13 ^b^	0.000
2-n-Heptylfuran	7.23 ± 2.83 ^a^	0.42 ± 0.23 ^b^	0.35 ± 0.16 ^b^	0.56 ± 0.27 ^b^	0.000
2-Butylfuran	8.95 ± 1.67 ^a^	1.49 ± 0.57 ^b^	0.29 ± 0.15 ^c^	1.11 ± 0.84 ^bc^	0.000
Ethylbenzene	10.79 ± 1.72 ^a^	5.08 ± 0.47 ^b^	3.06 ± 0.49 ^c^	4.28 ± 0.64 ^b^	0.000
Benzonitrile	7.71 ± 0.58 ^a^	1.60 ± 0.35 ^b^	2.02 ± 0.05 ^bc^	2.30 ± 0.31 ^c^	0.000
p-Xylene	12.62 ± 1.50 ^a^	6.38 ± 0.35 ^b^	4.11 ± 0.14 ^c^	4.77 ± 0.60 ^c^	0.000
Toluene	12.72 ± 0.66 ^a^	8.39 ± 0.26 ^b^	8.98 ± 0.29 ^c^	9.07 ± 0.35 ^c^	0.000
2-Hexylthiophene	3.78 ± 0.62	2.39 ± 0.41	-	-	0.001
2-Pentylpyridine,	11.99 ± 2.76	0.56 ± 0.31	-	-	0.000
Benzene	7.55 ± 0.76 ^a^	0.64 ± 0.29 ^b^	1.11 ± 0.48 ^c^	1.58 ± 0.40 ^c^	0.000
o-Xylene	12.27 ± 1.89 ^a^	5.87 ± 0.42 ^b^	3.37 ± 0.13 ^c^	3.95 ± 0.59 ^c^	0.000
Styrene	10.34 ± 0.63 ^a^	4.40 ± 0.83 ^b^	3.94 ± 1.82 ^b^	7.49 ± 1.06 ^c^	0.000
p-Cresol	8.09 ± 0.60	-	2.66 ± 0.35	2.69 ± 0.37	0.000
Indole	7.42 ± 2.43	-	1.09 ± 0.65	1.93 ± 0.48	0.000
2-Acety thiazole	5.30 ± 2.73 ^a^	2.52 ± 0.43 ^b^	4.64 ± 1.18 ^a^	5.10 ± 1.00 ^a^	0.024
2-Acetyl-2-thiazoline	10.95 ± 3.62 ^a^	5.16 ± 1.03 ^b^	4.00 ± 1.23 ^b^	3.93 ± 2.11 ^b^	0.000
Trisulfide, dimethyl	7.60 ± 2.23 ^a^	0.88 ± 0.31 ^b^	3.53 ± 0.36 ^c^	4.29 ± 0.69 ^c^	0.000
trans-Anethole	10.27 ± 1.41 ^a^	0.99 ± 0.94 ^b^	0.60 ± 0.80 ^b^	2.44 ± 0.68 ^c^	0.000
Indane	8.17 ± 0.87 ^a^	2.51 ± 0.15 ^b^	1.97 ± 0.23 ^b^	2.87 ± 0.26 ^bc^	0.000
Disulfide, dimethyl	5.41 ± 2.92 ^a^	1.03 ± 0.42 ^b^	5.45 ± 0.39 ^a^	5.58 ± 0.60 ^a^	0.000
N,N-Dibutylformamide	10.62 ± 2.94 ^a^	2.99 ± 0.56 ^b^	4.11 ± 1.06 ^b^	3.69 ± 0.60 ^b^	0.000

Values are presented as mean ± SD (*n* = 6). ^a–c^ Means within a row with different superscripts differ significantly (*p* < 0.05). DBS: Dabieshan cattle; LWB: local water buffalo; SM: Simmental; AG: Angus.

## Data Availability

The original contributions presented in the study are included in the article; further inquiries can be directed to the corresponding authors.

## Abbreviations

The following abbreviations are used in this manuscript:

GC × GC-TOF MS	Gas Chromatography-Time-of-Flight Mass Spectrometry
LD	Longissimus Dorsi
PH	Potential of Hydrogen
SFA	Saturated Fatty Acid
UFA	Unsaturated Fatty Acid
MUFA	Monounsaturated Fatty Acid
PUFA	Polyunsaturated Fatty Acid
EAA	Essential Amino Acid
NEAA	Non-Essential Amino Acid
